# Clinical Lectures

**Published:** 1867

**Authors:** Wm. C. Lyman

**Affiliations:** Late Surgeon U.S. Navy, and now Resident Physician U.S. Marine Hospital, Chicago


*Clinical Lectures.* By Wm. C. Lyman, M.D., late Surgeon U.S. Navy, and now Resident Physician U.S. Marine Hospital, Chicago.

Gentlemen:—The case that occupies our attention this morning is one of *necrosis* of the lower third of the tibia. Necrosis, as you well know, is death of a bone, and is entirely synonymous in meaning with mortification or gangrene, when those terms are applied to death of any part of one of the soft tissues of the body.

This patient was admitted into the hospital three months ago, and at that time presented a train of symptoms which we will endeavor to give to you, in connection with a short history of the case, as given by the patient himself. He is a native of the United States, 24 years of age, and, as far as can be ascertained, without any hereditary constitutional taint. The first symptom of disease in the part affected, was first observed 12 years ago. It was acute periostitis, or ostitis, the result of exposure to cold. A period of hardship, incident to his life as a sailor, was followed by the usual concomitant symptoms of inflammation of bone and its fibrous covering. During these 12 years, the part has never been in a condition of health, a degree of chronic inflammation being always present, except at such times as it became acute from constantly renewed exposure. Each winter has sufficed to induce an attack which has always sent him to a hospital.

The symptoms presented on his admission were sufficiently characteristic to be at once recognized. The most prominent among these were the great swelling of the limb, with a partial atrophy both above and below the seat of the disease; the presence of several little papilla-like prominences, each one being the opening of a sinus leading to the dead bone, with continual discharge of sanious pus; and the unmistakable presence of dead bone on introducing a probe into the sinus, the sensation of which, being once experienced, is ever after easily recognized. There was, also, a decidedly marked impairment of the neighboring joints.

His general condition was that of extreme exhaustion—there was hectic fever, colliquative diarrhœa and perspiration, and great emaciation. His mental condition was truly deplorable—his countenance exhibited the greatest anxiety, he would burst into tears at the slightest opposition to his wishes, was peevish, restless, and sleepless in the extreme. It is probable, in this case, that that which began as periostitis, twelve years ago, has progressed, by exposure to additional causes and perpetuation of the original disease, to ostitis, and, finally, osteo-myelitis, resulting in the complete destruction of the periosteum, osseous tissue, endosteum, and medullary substance.

If we look closely into the causes of this disease, we shall find that the form that may be called idiopathic commonly arises in young persons, who have well-marked strumous diathesis, and in whom the vital power is low, and who are, consequently, more obnoxious to diseases of any character, from some exposure to cold, as a plunge in cold water, or getting wet in a shower. These may be followed, in a few days, by rigors, febrile movement, pain in the part, and the usual train of symptoms attending acute inflammation. It has also occurred without apparent cause, and has followed idiopathic fever. Syphilis, either hereditary or acquired, is also a prolific cause, but its seat is more frequently in the cranial and nasal bones. The abuse of mercury may also be mentioned, that potent drug being as powerful for evil when injudiciously administered, as it is for good when given with due caution. That exposure to the fumes of phosphorus which unavoidably occurs in the manufacture of matches, is often followed by necrosis of the alveolar processes. The rheumatic and scorbutic diatheses are, undoubtedly, occasional producing causes. Among the traumatic causes, are resections, amputations, compound fractures, gunshot wounds, contusions, and transportation of wounded. The latter is, necessarily, rendered a fruitful cause by the consequent fatigue, excitement, and pain, the absence of proper medicines, food, and shelter, and the resulting mental depression that wields such a powerful influence over the wounded soldier when he feels neglected.

The abundant experience of our army surgeons in the late war, furnishes many examples which go to prove the truth of such assertions. Impure air, food, and water are well-known causes of destructive inflammation of bone in patients exposed to such influences after any surgical operation; these conditions are always worthy of our attention, particularly in military practice, where hospitals are often crowded, and the worst results follow any neglect, even for a few hours. As such causes can almost always be guarded against, they should never be permitted to add to the fatality of this or any other disease.

The statistics of this disease, which cannot well be detailed here, show that a large majority of the cases occur among young people—probably the period between the 20th and 30th years is the time that this disease is most frequently observed. It is also equally well established by the observations upon this point, that males are much more liable than females to be the subjects of this malady. The reasons for this difference are not so easily understood; the greater amount of exposure to general causes of disease, and of accidents, hardly account for the difference, which may be estimated as 5 to 1.

In its extent, necrosis may be partial or general. If the entire bone is in a necrosed condition, it is usually from a succession of attacks and gradual extension of the disease, and does not involve the whole of it at once. Occasionally, it extends into the structure of a joint, when both local and general symptoms are aggravated, and the danger of a fatal result proportionately increased. Instead of involving the whole diameter of the shaft of a bone, it may only affect the outer part, causing a portion to scale off. This is called "exfoliation;" where there has been a deposit of new osseous tissue surrounding and enclosing the old, it is called a "sequestrum." The operations appropriate in the two cases differ materially, although the one result, the removal of the decayed mass, is its object. The removal of an exfoliation requires the exposure of the bone, and the cutting away of the dead portion with the chisel or gouge. The removal of a sequestrum requires the opening not only of the muscular structure, but an opening in the newly formed bone itself, which will admit of its passage through. The saw, trephine, forceps, and chisel are variously used, as may be required.

The reparatory process, in cases of this kind, is interesting to contemplate. By the action of the absorbents, detachment and ultimate expulsion of the dead bone is begun, and simultaneously the capillary vessels begin to deposit around material for the creation of the new structure. This occurs around the bone, and in all parts where the periosteum remains intact, it assumes the appearance of cartilage, which, as the process goes on, presents points of ossification, from which the change progresses in the same manner as in the original bone. The new structure acquiring thickness and density in a gradual manner, which it may require several weeks or months to accomplish, each case progressing according to the general vigor that the individual may possess. The new tissue usually becomes more dense and hard, and contains more earthy matter than the original, and is usually larger, thus compensating in increase of size and strength for any loss of power by loss of symmetry. That the agent of reproduction is the periosteum, and not the endosteum, is evident, from the latter being in that part always destroyed; that under other circumstances it might be an agent of epigenesis is probable. Granulations spring up from the surface of bone that may be denuded of periosteum, yet remain closely enough connected with the lining tissue to retain its vitality, and thus assist in the reconstruction process.

The new growth is always pierced by a number of small openings, varying from the smallest rounded opening to a fissure of considerable size. It is a fact of some practical value, that these openings are always in that part where the least tissue covers the bone, and whatever their number and direction, they always communicate with the necrosed bone itself, affording exit for the purulent matter.

There has been a great deal of difference of opinion among pathologists, relative to the manner of reproduction of a bone. It has been warmly asserted that it can be produced by granulations from each end of the bone, after a part has been removed, without the agency of the periosteum. It may not yet be definitely settled, but practically, it is found that the better the periosteum is preserved, the more complete and useful will be the new growth. While it appears that a bone cannot be reproduced when a portion has been entirely removed, it is certain that a very small amount of bony connection subserves the the purpose of reproduction of nearly the whole organ.

It was evident from the appearance of this patient, that surgical interference was immediately necessary. The only question that presented itself was, whether the limb should be at once amputated, or this mass of dead matter be removed. As there was no sequestrum in this case, no ossific deposit having occurred around the dead portion, it appeared as if the removal of the dead bone would leave a vacant space of full two inches between the sound portions, leaving only the fibula to sustain the weight of the body. With the idea of saving that part, if possible, it was decided to remove the dead bone, and amputate subsequently, if it could not be avoided.

The operation was at once proceeded with—a longitudinal incision of four inches in length, along the surface of the tibia, crossed by one of three inches, and turning up of the two flaps thus made, sufficiently exposed the part; the dead bone was immediately removed with the chisel. It was here discovered that a portion of the "external border" of the tibia was still living, although a sinus passed between it and the fibula. In its smallest part it was rounded, and not more than one-quarter of an inch in diameter. Being at the bottom of the wound, it was difficult to tell whether or not there was any periosteum remaining. On this slender basis, our hopes of the future integrity of the limb were founded. The diseased portion having been fully removed, the flaps were replaced and the operation finished. The treatment, since the operation, has been simply water dressing or simple cerate. He had liberal diet, alcoholic stimulants at night, for a few days.

We have had the satisfaction of observing no return of the hectic, sweating, or diarrhœa; his mental condition rapidly improved, his cheerfulness returning with the confirmation of his hope of ultimate recovery. Profuse granulations appeared on all sides of the chasm left after the removal of the bone, not only on the soft parts but on the cut surfaces of the tibia itself, and upon the slender isthmus of bone between the portions of the shaft. We have been gratified by seeing this opening gradually fill up with osseous material, and at the present time the patient walks with perfect ease and safety to the limb, which is, to all appearance, perfectly sound and strong.

This case may be considered chiefly remarkable for the slender connection left between the parts of the tibia, furnishing an exceedingly small amount of periosteum as an epigenetic agent for the new osseous growth. It is probable that without this slender isthmus of bone, how ever well the remaining extremities may have performed their duty, there would have been lacking that nucleus for the deposit of ossific material that would insure a useful limb. There is no doubt, that "points of ossification" are as necessary in the reproduction of a bone as they are in its original development.

